# The ADAM9/WISP-1 axis cooperates with osteoblasts to stimulate primary prostate tumor growth and metastasis

**DOI:** 10.7150/ijbs.77495

**Published:** 2023-01-01

**Authors:** An-Chen Chang, Liang-Wei Lin, Yen-Chen Chen, Po-Chun Chen, Shan-Chi Liu, Huai-Ching Tai, Hsi-Chin Wu, Shian-Ying Sung, Tien-Huang Lin, Chih-Hsin Tang

**Affiliations:** 1 Translational Medicine Center, Shin Kong Wu Ho-Su Memorial Hospital, Taipei, Taiwan.; 2 Graduate Institute of Biomedical Sciences, China Medical University, Taichung, Taiwan.; 3 Department of Life Science, National Taiwan Normal University, Taipei, Taiwan.; 4 Department of Medical Research, China Medical University Hospital, China Medical University, Taichung, Taiwan.; 5 Department of Medical Education and Research, China Medical University Beigang Hospital, Yunlin, Taiwan.; 6 School of Medicine, Fu-Jen Catholic University, New Taipei City, Taiwan.; 7 Department of Urology, Fu-Jen Catholic University Hospital, New Taipei City, Taiwan.; 8 School of Medicine, China Medical University, Taichung, Taiwan.; 9 Department of Urology, China Medical University Hospital, Taichung, Taiwan.; 10 Department of Urology, China Medical University Beigang Hospital, Beigang, Yunlin, Taiwan.; 11 Graduate Institute of Clinical Medicine, School of Medicine, College of Medicine, Taipei Medical University, Taipei, Taiwan.; 12 International Ph.D. Program for Translational Science, College of Medical Science and Technology, Taipei Medical University, Taipei, Taiwan.; 13 The Ph.D. Program for Translational Medicine, College of Medical Science and Technology, Taipei Medical University, Taipei, Taiwan.; 14 TMU Research Center of Cancer Translational Medicine, Taipei Medical University, Taipei, Taiwan.; 15 Office of Human Research, Taipei Medical University, Taipei, Taiwan.; 16 TMU-Research Center of Urology and Kidney, Taipei Medical University, Taipei, Taiwan.; 17 Department of Urology, Buddhist Tzu Chi General Hospital Taichung Branch, Taichung, Taiwan.; 18 School of Post-Baccalaureate Chinese Medicine, Tzu Chi University, Hualien, Taiwan.; 19 Chinese Medicine Research Center, China Medical University, Taichung, Taiwan.; 20 Department of Biotechnology, Asia University, Taichung, Taiwan.

**Keywords:** Prostate cancer, Osteoblast, WISP-1, ADAM9, Metastasis

## Abstract

**Background:** Metastatic prostate cancer (PCa) predicts a poor prognosis and lower likelihood of survival. Osteoblasts (OBs) are known to be responsible for the synthesis and mineralization of bone, although it is unclear as to whether PCa in the prostate gland cooperates with OBs in bone to promote PCa malignant transformation. We aimed to elucidate how primary PCa cells cooperate with distal OBs and contribute to the vicious cycle that leads to metastatic PCa.

**Methods:** N-cadherin, E-cadherin, and Twist protein expression were measured by Western blot. Twist translocation into the nucleus was detected by the immunofluorescence (IF) assay. Enzyme-linked immunosorbent assay (ELISA) detected protein levels in human serum samples. Levels of candidate protein expression were examined by the human cytokine array. Prostate tumor growth and metastasis were analyzed by orthotopic and metastatic prostate cancer models, respectively. Immunohistochemistry (IHC) staining was used to observe ADAM metallopeptidase domain 9 (ADAM9) and WNT1 inducible signaling pathway protein 1 (WISP-1) expression in tissue.

**Results:** Our *in vitro* and *in vivo* analyses have now discovered that primary PCa expressing ADAM9 protein enables the transformation of OBs into PCa-associated osteoblasts (PCa-OBs), inducing WISP-1 secretion from PCa-OBs in the bone microenvironment. The upregulation of WISP-1 in bone provided feedback to primary PCa and promoted PCa cell aggressiveness via epithelial-mesenchymal transition (EMT) activity. Elevated levels of WISP-1 expression were detected in the serum of patients with PCa. ADAM9 levels were overexpressed in tumor tissue from PCa patients; ADAM9 blockade interrupted OB-induced release of WISP-1 and also suppressed primary tumor growth and distal metastasis in orthotopic PCa mouse models.

**Conclusion:** Our study suggests that the ADAM9/WISP-1 axis assists with metastatic PCa progression. Thus, targeting the ADAM9/WISP-1 axis may help to prevent the malignant phenotypes of PCa cells.

## Introduction

Prostate cancer (PCa) commonly affects the urinary tract of men, whose susceptibility for this cancer increases with age 1. Around 77% of newly diagnosed PCa cases present with localized disease; 11% and 5% of cases present with disease that has metastasized to the regional lymph nodes and distant metastasis, respectively 2. Metastatic PCa has a low 5-year survival rate of 30%, compared with nearly 100% for local or regional disease 3. A clear understanding of the mechanism of malignant progression in PCa would enable researchers to develop early prevention and intervention strategies.

Secretion of androgen by testicular Leydig cells plays a pivotal role in prostate epithelial cell growth and differentiation 4 and PCa development 5. The requirement for androgen in PCa progression has led to the development of androgen deprivation therapy (ADT) by orchiectomy or luteinizing hormone-releasing hormone (LHRH) agonists and LHRH antagonists 6. However, ADT is not ideal, because patients typically develop castration-resistant prostate cancer that progresses to metastatic disease, with a poor survival time of only 16-18 months 7, 8. An alternative approach is needed to prevent and manage PCa progression. The growth factor-rich environment in bone increases tumor growth 9. The finding that PCa-derived extracellular vesicles support the formation of a premetastatic niche in bone suggests that the primary PCa cooperates with bone to regulate future metastasis in distal bone 10. However, it remains unclear as to whether bone cell-derived factors initiate the malignant transformation of primary PCa cells and stimulate the development of metastases.

Osteoblasts (OBs) play a critical role in the repair and regeneration of the bone matrix 11. Growing evidence suggests that OBs release a range of different molecules including bone morphogenic proteins (BMPs), alkaline phosphatase (ALP), osteopontin (OPN) that stimulates osteoblastogenesis 12, 13, and also vascular endothelial growth factor A (VEGF-A) and hypoxia-inducible factor 1-alpha (HIF-1α), which both regulate bone marrow angiogenesis 14-16. OBs also produce WISP-1 to drive new sites of bone formation during embryonic development 17. WISP-1 reportedly functions as an oncogene by mediating the transformation of normal kidney fibroblasts into tumors *in vivo* 18. Overexpression of WISP-1 promotes the motility and invasiveness of cancer cells 19, 20, triggers EMT activity 21, and enhances experimental tumorigenesis and distal metastasis 20, 22. Moreover, WISP-1 expression correlates positively with clinical staging, lymphatic metastasis and poor clinical outcomes in human cancers 23, 24, although it is not yet known whether OB-derived WISP-1 facilitates the malignant transformation of primary PCa tumors.

In this study, our data indicate that primary PCa cells may distally influence OBs in bone, transforming OBs into PCa-OBs, with subsequent increases in WISP-1 expression in the bone microenvironment. Positive feedback from PCa-OBs expressing WISP-1 elevates the invasiveness of primary PCa cells via EMT functioning. WISP-1 enhances EMT activity in PCa cells by suppressing the epithelial marker E-cadherin and inducing the mesenchymal markers N-cadherin and Twist. Cytokine array analysis revealed that soluble ADAM9 (ADAM9-S) is a main mediator secreted from primary PCa cells, capable of regulating PCa-OB formation and WISP-1 expression. Higher ADAM9 levels were detected in clinical PCa tumor tissue compared with normal tissue specimens, while ADAM9 blockade suppressed PCa tumor growth and reduced the formation of distant metastasis in orthotopic PCa mouse models. Our results not only elucidate the mechanism underlying cooperation between primary PCa tumor cells and distal OBs, but also indicate that targeting the ADAM9/WISP-1 axis may interrupt the progression to metastatic disease.

## Materials and Methods

### Cell culture

The human osteoblastic-like cell line MG-63 was grown in DMEM medium. The human prostate epithelial cell line (PZ-HPV-7) was grown in keratinocyte serum-free medium supplemented with bovine pituitary extract. All PCa cell lines (LNCaP, PC-3 and DU145) were grown in RPMI-1640 medium. All cell types were cultivated at 37°C in a 5% CO_2_ atmosphere.

### Collection of conditioned medium (CM)

MG-63 cells, human prostate epithelial cells (PZ-HPV-7), and PCa cell lines (LNCaP, PC-3 and DU145) were seeded (at a rate of 2x10^6^/10 mL each) in a 10 cm dish with fetal bovine serum (FBS) for 24 h, then the medium was replaced with serum-free medium. After 48 h, CMs were collected as osteoblast CM (OBCM), PZ-HPV-7 CM, LNCaP CM, PC-3 CM, and DU145 CM.

**Figure 1:**
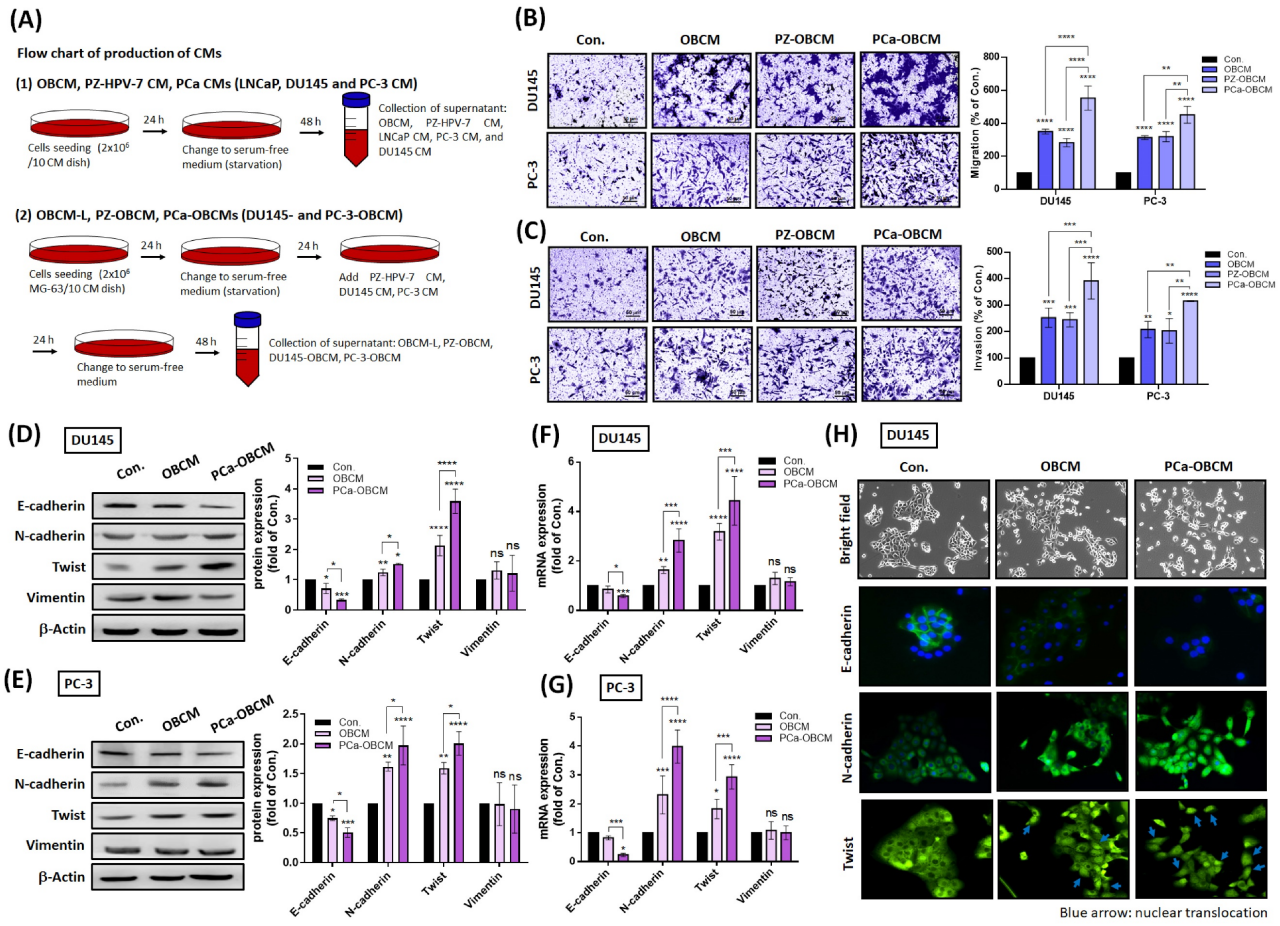
** PCa cells corporate with osteoblasts to facilitate invasiveness.** (A) A schematic diagram showing the collection of different CMs from OBs, PCa cells, and PCa-OBs. (B&C) Treatment of PC-3 and DU145 cells with OBCM, PZ-OBCM or PCa-OBCM for 24 h. The Transwell assay examined cell migration and invasion activities. (D-G) PCa cells were incubated with OBCM or PCa-OBCM for 24 h, then examined for EMT markers using Western blot and qRT-PCR assays. ImageJ software quantified levels of EMT protein marker expression. (H) The cell scattering phenotype was imaged by bright-field microscopy. Levels of E-cadherin, N-cadherin and Twist translocation into the nucleus were examined by the immunofluorescence (IF) assay. The nuclear location of DU145 cells was determined by DAPI staining. Blue arrow: nuclear translocation of Twist. All data are expressed as the mean ± the S.D. of three samples. *P < 0.05, **P < 0.01, ***P < 0.001, ****P < 0.0001.

In another series of experiments, MG-63 cells (2x10^6^) were seeded in a 10 cm dish for 24 h, then incubated with serum-free medium for another 24 h. The cells were treated with 30% PZ-HPV-7 (PZ) CM, PC-3 CM, or DU145 CM for 24 h, then incubated in serum-free medium for 48 h. CMs were collected as PZ-OBCM, PC-3-OBCM, and DU145-OBCM. Long-term OBCM (OBCM-L) was also collected without PCa CM pretreatment (Figure 1A).

### Analysis of ADAM9 expression in PCa patients

Correlations between ADAM9 expression and promoter methylation levels for normal and tumor tissues were analyzed using The Cancer Genome Atlas (TCGA), Oncomine and UALCAN tools.

### In vitro cell migration and invasion assay

Cell migratory and invasive activity was evaluated through Transwell inserts in 24-well dishes (Costar, NY, USA), as according to our previous research 25. Migratory and invasive cells were imaged under ×200 magnification using an Eclipse Ti2 microscope (Nikon, Tokyo, Japan).

### Quantitative real-time polymerase chain reaction (qRT-PCR) assay

A Magic RT Master Mix cDNA synthesis kit (Bio-Genesis Technologies, lnc., Hsinchu, Taiwan) was used to convert cDNA from RNA fragment, according to the manufacturer's recommendations. Sequence-specific primers (N-cadherin, E-cadherin, Twist, vimentin, and GAPDH) and Taqman one-step PCR Master Mix (Applied Biosystems, CA, USA) were used to perform qRT-PCR analysis, as according to our previous study 26.

### Human cytokine array

A human cytokine array (R&D Systems, MN, USA) containing a total of 32 different cytokine antibodies spotted in duplicate on two membranes was used to analyze protein expression in CM, according to the manufacturer's recommendations. Levels of 32 proteins were detected through enhanced chemiluminescence and quantified by UN-SCAN-IT gel 6.1 software.

### Analysis of ADAM9 protein levels in CMs by ELISA assay

The ADAM9 ELISA kit (PeproTech, NJ, USA) assayed ADAM9 protein concentrations in CM, according to the manufacturer's instructions.

### Orthotopic and metastatic prostate cancer models

Six‐week‐old male nude mice, bought from BioLASCO Taiwan Co., Ltd. (Taipei, Taiwan), were injected in the anterior prostate with 5 × 10^5^ PC-3 cells suspended in 50 μL Matrigel via a 22-gauge needle. Tumor development and distal metastasis was monitored using the IVIS Spectrum scanner (Xenogen, AZ, USA). After 2 weeks, the mice in the orthotopic model (n=7) were humanely sacrificed in a CO_2_ chamber. The tumors and tibias were collected before immunostaining. Mice in the metastatic model (n=9) were humanely sacrificed after 5 weeks and distant organs, including the liver and lungs, were harvested. Hematoxylin and eosin (H&E) staining was used to investigate distal metastasis in liver and lung specimens.

### Ethics statement

All procedures in the animal studies were approved by the Institutional Animal Care and Use Committee and performed according to the Guidelines of Animal Experimentation issued by Shin Kong Wu Ho-Su Memorial Hospital (Approval No: 109MOST0016). Serum samples were collected from 11 patients undergoing surgical resection in China Medical University Hospital, under approval granted by the Institutional Review Board of China Medical University Hospital (Approval No: CMUH109-REC2-142). Written informed consent was obtained from each study participant before enrollment.

### Statistical analysis

A statistical comparison between two samples was performed using the Student's *t*-test. One-way ANOVA followed by Bonferroni's *post hoc* comparison tests compared the means of more than two groups. The results are reported using means ± standard deviations. Statistical significance was indicated if P < 0.05.

## Results

### PCa cells collaborate with OBs to promote cell invasiveness via EMT induction

In order to determine whether primary PCa cells cooperate with OBs to promote the malignant transformation of PCa, samples of PCa CM, OBCM, PZ-OBCM and PCa-OBCM were collected for Transwell assay analyses of cancer cell motility and invasion (Fig. 1A). Our stimulation of PCa cell lines DU145 and PC-3 with PCa-OBCM revealed that PCa-OBCM significantly enhanced PCa cell migration and invasion compared with OBCM and PZ-OBCM treatment (Fig. 1B&C). As EMT is critical for tumor initiation, progression and metastasis 27, we examined whether EMT is involved in PCa-OB-induced mediation of PCa cell invasiveness. As shown in Fig. 1D-H, PCa-OBCM enhanced EMT function by upregulating N-cadherin and Twist expression, and suppressing E-cadherin expression; levels of vimentin, Snail, ZEB1, and ZEB2 expression were unchanged (Fig. 1D-G; Supplementary Fig. 1A&B). Moreover, nuclear translocation of the Twist transcription factor was observed after PCa-OBCM treatment (Fig. 1H). We also found that collection of OBCM under a prolonged period of time (OBCM-L; the same amount of collection time as for PCa-OBCM) did not further increase N-cadherin or Twist expression, or inhibit E-cadherin expression when compared with OBCM-induced values, suggesting that OBCM-induced EMT activation is limited in PCa cells (Supplementary Fig. 1C). These findings suggest that prolonged exposure to PCa-OBCM stimulates the EMT phenomenon and thus increases the invasiveness and aggressiveness of PCa cells.

### Primary PCa promotes WISP-1 expression in distal OBs according to in vitro and in vivo analyses

**Figure 2:**
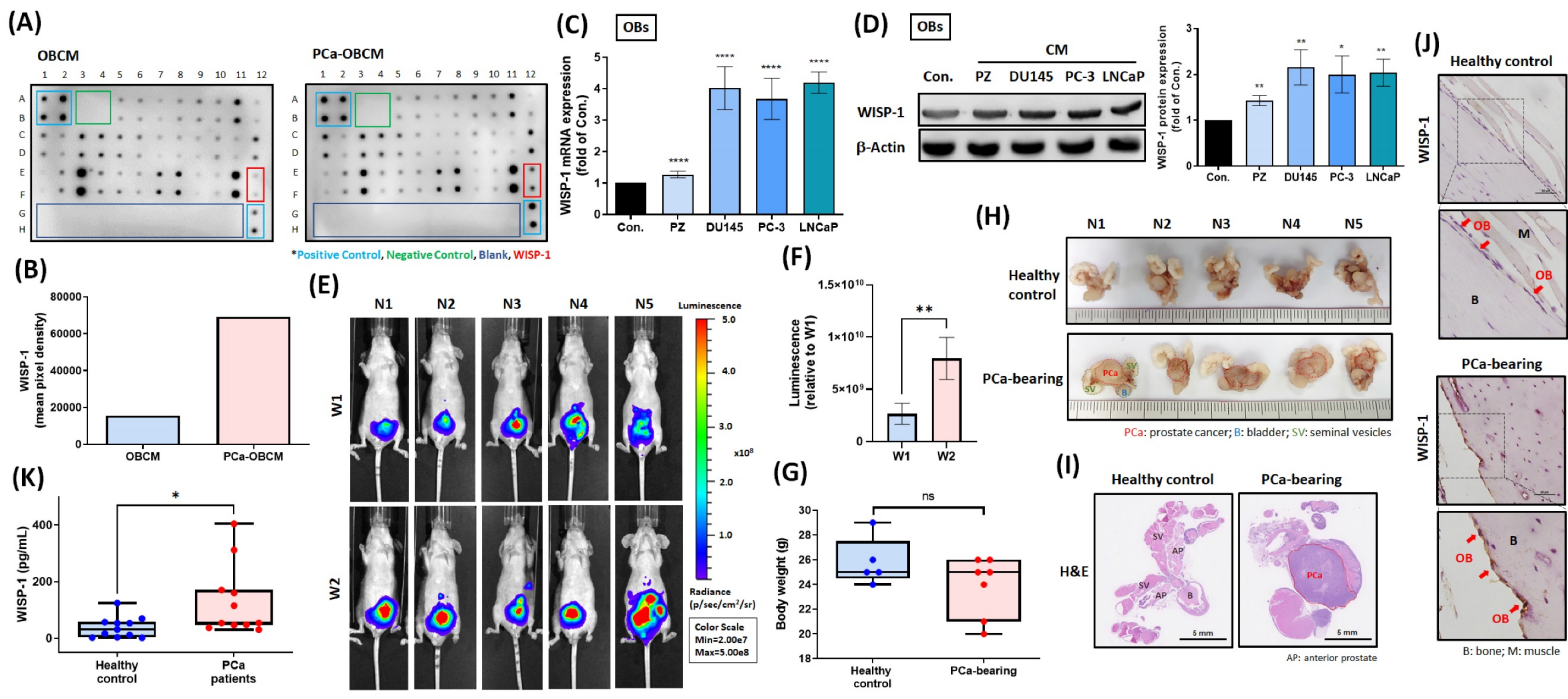
** Primary PCa tumors promote WISP-1 expression in distal OBs.** (A) Levels of candidate protein expression were examined by the cytokine array. (B) WISP-1 expression levels were quantified by UN-SCAN-IT gel 6.1. (C&D) Treatment of OBs with PZ CM or different PCa CMs (DU145, PC-3, LNCaP) for 24 h. WISP-1 mRNA and protein expression was measured using qRT-PCR and Western blot assays, respectively. ImageJ software quantified levels of WISP-1 protein expression. (E&F) Luminescence imaging of tumor-bearing mice was performed weekly using the IVIS spectrum system, and the luminescent intensity of photons emitted from each tumor was quantified. (G) Body weights for healthy controls and tumor-bearing mice were recorded manually at the end of week 2. (H) Images of excised tumors obtained by bright-field microscopy. (I) H&E-stained images from whole-mount tumor tissue. (J) IHC staining revealed WISP-1 expression in OBs from tibia specimens. Red arrows: OBs. (K) WISP-1 levels in healthy human serum and PCa serum were detected by ELISA. *P < 0.05, **P < 0.01, ***P < 0.001, ****P < 0.0001.

A cytokine array that examined levels of candidate protein secretion from PCa-OBs showed higher WISP-1 expression in PCa-OBCM compared with OBCM (Fig. 2A&B). Treatment of OBs with different PCa CMs (DU145, PC-3 and LNCaP) promoted WISP-1 mRNA and protein expression (Fig. 2C&D). Orthotopic PCa tumors were established for 2 weeks in nude mice to verify WISP-1 expression in distal OBs. IVIS imaging demonstrated that at 2 weeks, tumors were larger in size compared with tumors at 1 week (Fig. 2E&F); body weight was not affected by tumor growth (Fig. 2G). Orthotopic PCa tumors and healthy prostate glands were then removed and stained with H&E via whole-mount imaging to confirm tumor development (Fig. 2H&I). Histopathological examination of WISP-1 expression revealed higher levels in distal OBs located on the tibia bone surface in mice orthotopically implanted with PCa cell lines compared with mice that were not (Fig. 2J). Similarly, analyses of healthy human serum and PCa serum found higher WISP-1 expression in the PCa serum (Fig. 2K). Our investigations indicate that primary PCa tumors may influence the transformation of distal OBs into PCa-OBs capable of promoting WISP-1 secretion in the bone microenvironment.

### PCa-OB-derived WISP-1 confers aggressiveness in PCa cancer cells

**Figure 3:**
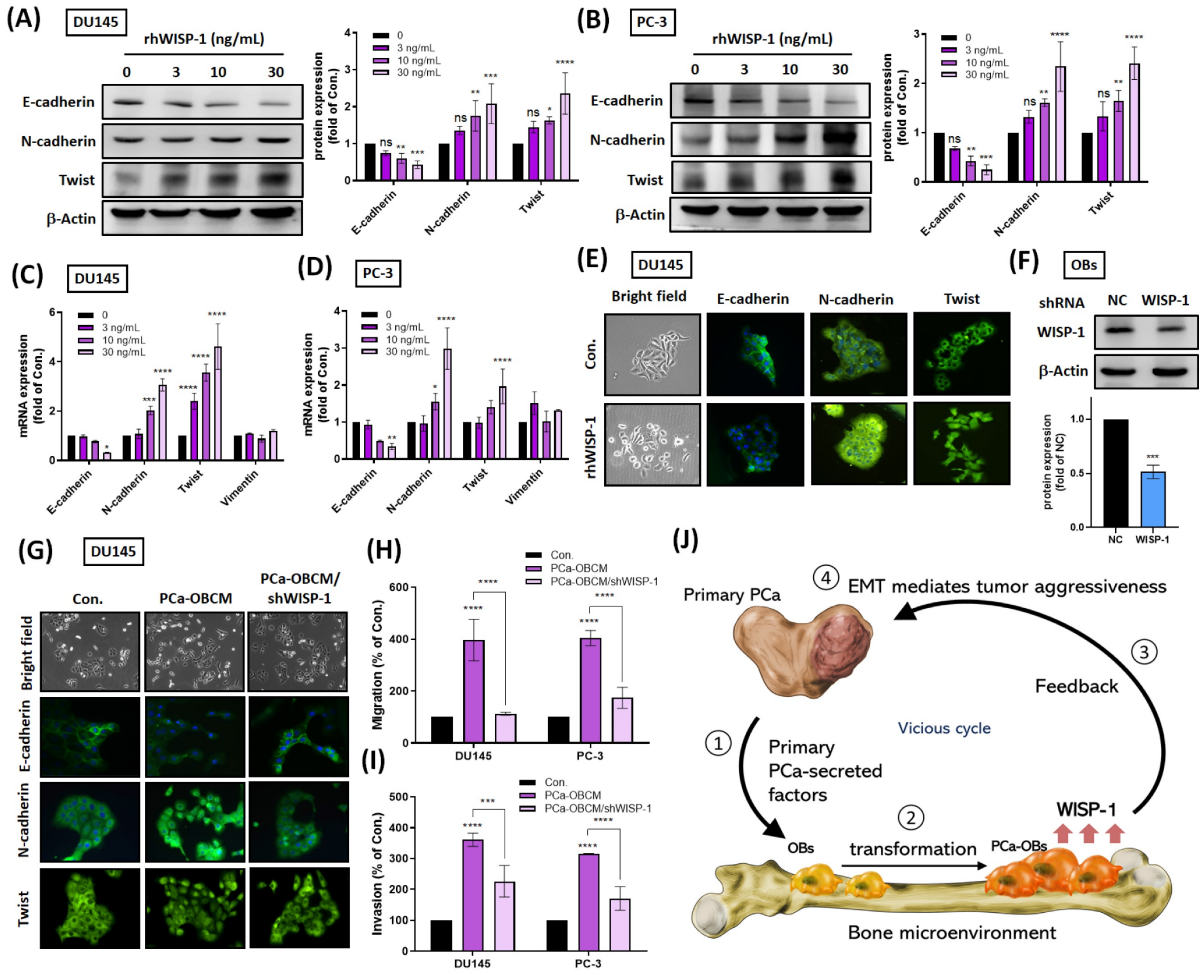
**PCa-OB-derived WISP-1 increases PCa cell invasiveness.** (A-D) After incubation of PCa cells with rhWISP-1 (0, 3, 10, or 30 ng/mL) for 24 h, levels of E-cadherin, N-cadherin and Twist protein were quantified by Western blot and ImageJ software, respectively; mRNA expression was analyzed by qRT-PCR. (E&G) DU145 cells were stimulated with rhWISP-1 (30 ng/mL), PCa-OBCM, or PCa-OBCM/shWISP-1 for 24 h, before imaging the cell scattering phenotypes by microscope. Levels of E-cadherin, N-cadherin and Twist translocation into the nucleus were examined by the IF assay. (F) The knockdown efficiency of WISP-1 shRNA on OBs was quantified by Western blot and ImageJ software, respectively. (H&I) The Transwell assay examined levels of PCa cell migration and invasion after PCa-OBCM or PCa-OBCM/shWISP-1 treatment for 24 h. (J) Modeling shows how primary PCa cooperates with distal PCa-OBs to constitute a vicious cycle with increasing PCa aggressiveness via EMT activation. *P < 0.05, **P < 0.01, ***P < 0.001, ****P < 0.0001.

To examine WISP-1 protumor functions, recombinant human WISP-1 (rhWISP-1) protein was used to measure EMT function in PCa cells. We observed that rhWISP-1 treatment elevated N-cadherin and Twist expression and reduced E-cadherin expression in PCa cell lines DU145 and PC-3 (Fig. 3A-E). Immunofluorescent staining revealed that rhWISP-1 promoted the translocation of Twist into the nucleus (Fig. 3E). Incubating PCa cells with PCa-OBCM promoted EMT activity, cell migration and invasion, while knockdown WISP-1 inhibited PCa-OBCM-mediated invasiveness in OBs (Fig. 3F-I). It appears that PCa-OBs regulate cancer cell invasiveness via WISP-1 upregulation and that WISP-1 blockade in OBs reduces this phenomenon. The schematic diagram in Figure 3J summarizes the results from Figures 1-3, showing how primary PCa promotes the transformation of distal OBs into PCa-OBs and is accompanied by increasing levels of WISP-1 expression in the bone microenvironment, subsequently triggering PCa cell migratory and invasive activities via EMT activation.

### ADAM9 supports the formation of a vicious cycle between primary PCa cells and OBs

**Figure 4:**
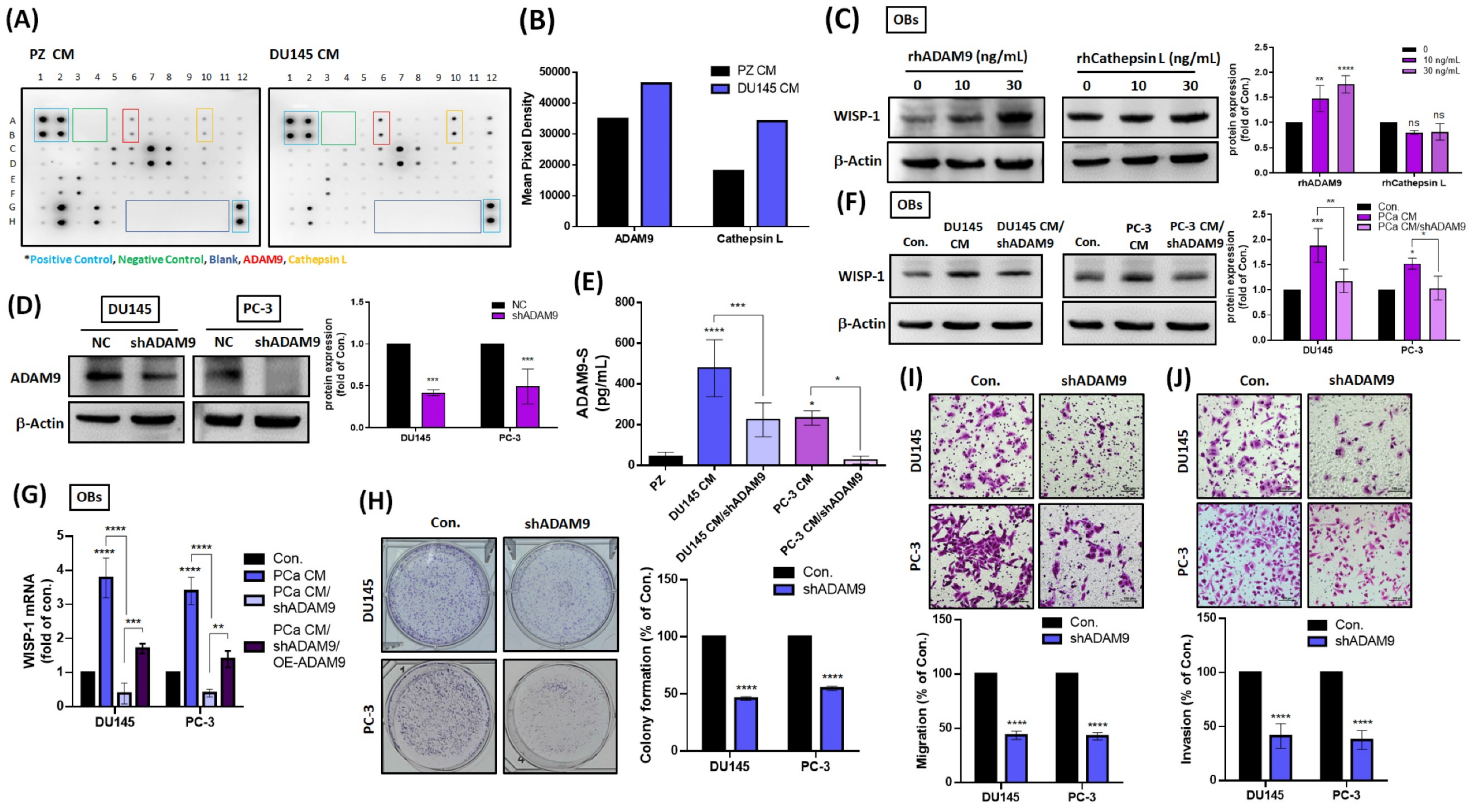
**ADAM9 is responsible for the initiation of a vicious cycle between primary PCa and OBs.** (A) Cytokine arrays were used to analyze levels of candidate proteins. (B) Levels of ADAM9 expression were quantified by UN-SCAN-IT gel 6.1. (C) PCa cells were stimulated with rhADAM9 (0, 10, or 30 ng/mL) and rhCathepsin L (0, 10, or 30 ng/mL) for 24 h, then WISP-1 protein levels were determined using the Western blot assay. (D) The knockdown efficiency of ADAM9 shRNA upon both DU145 and PC-3 cells was determined by Western blot. (E) DU145 and PC-3 cells were transfected with or without ADAM9 shRNA (1 µg/µL) for 24 h, then CM was collected and quantified for ADAM9-S levels by ELISA. (F) PCa CMs and PCa CM/shADAM9 were added to OBs for 24 h, then examined for WISP-1 protein by Western blot. (G) OBs were treated with PCa CMs, PCa CM/shADAM9, or PCa CM/shADAM9/OE-ADAM9 for 24 h. WISP-1 mRNA expression was analyzed by qRT-PCR assays. (H-J) PCa cells were transfected with ADAM9 shRNA (1 µg/µL) to observe colony formation, migratory and invasive activity. *P < 0.05, **P < 0.01, ***P < 0.001, ****P < 0.0001.

Next, we sought to determine what factors are released by primary PCa cells that initiate crosstalk between primary PCa cells and distal OBs. Cytokine array data revealed that the soluble forms of ADAM9 (ADAM9-S) and cathepsin L (cathepsin L-S) levels were elevated in DU145 CM compared with PZ CM (Fig. 4A&B). Treatment of OBs with different concentrations of rhADAM9 dose-dependently increased WISP-1 protein expression, whereas rhCathepsin L had no significant effect (Fig. 4C). To investigate whether the amount of ADAM9-S secreted from PCa cells affects WISP-1 expression in OBs, subsequent experiments quantified shRNA-mediated depletion of ADAM9 in PCa cells. Firstly, total ADAM9 protein and ADAM9-S levels were detected by Western blot and ELISA assays, respectively, to confirm the efficiency of ADAM9 depletion in PCa cells (Fig. 4D&E). CM from PCa cells with or without ADAM9 depletion was collected and incubated with OBs to measure WISP-1 expression. The resulting data showed that ADAM9 depletion suppressed PCa-induced increases in WISP-1 protein and mRNA expression (Fig. 4F&G), while ADAM9 overexpression (OE-ADAM9) rescued WISP-1 mRNA expression in OBs (Fig. 4G). ADAM9 depletion also interrupted colony formation, cell motility and invasiveness of PCa cells (Fig. 4H-J).

**Figure 5:**
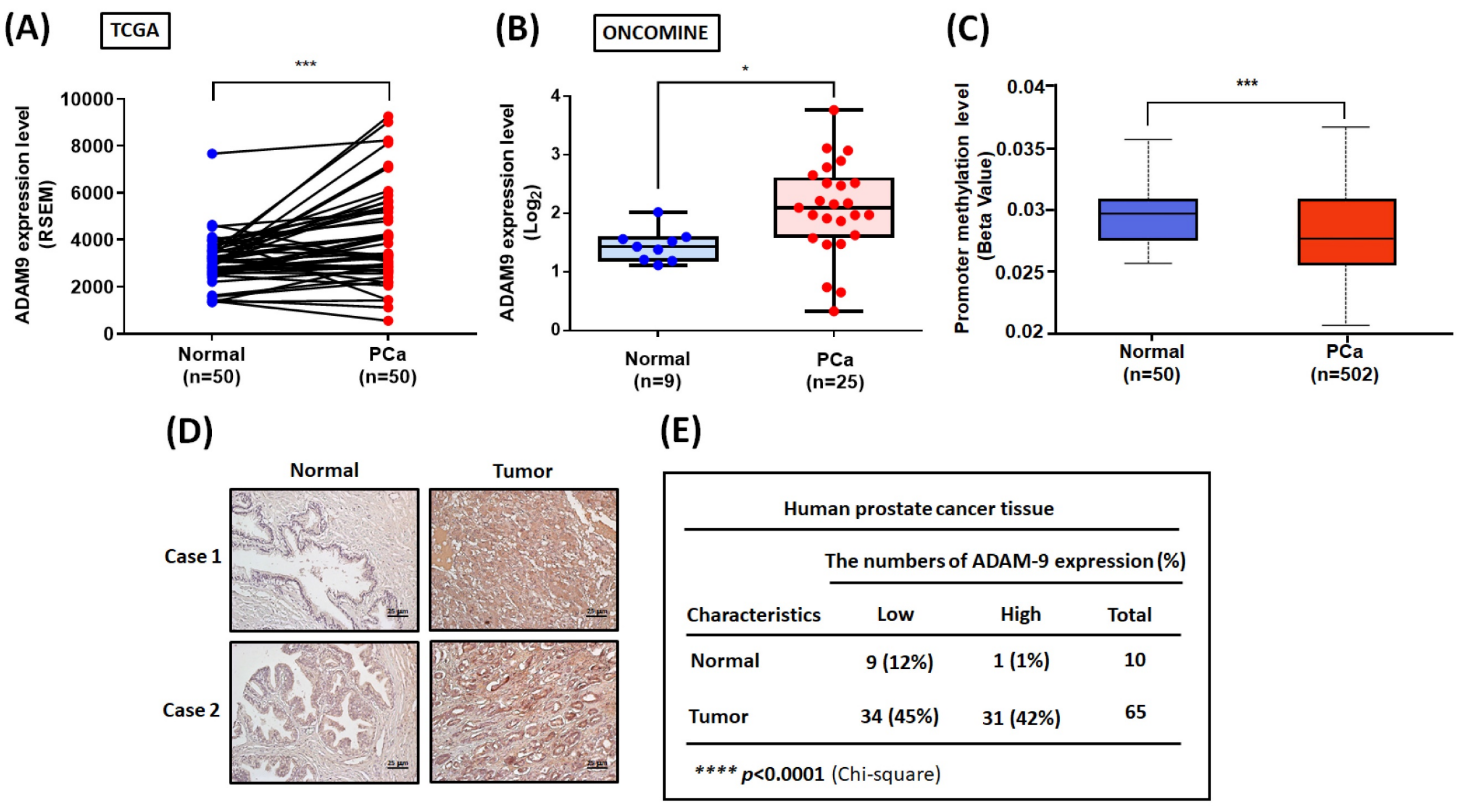
** ADAM9 expression in human PCa tissue.** (A) Levels of ADAM9 expression in paired PCa and adjacent normal tissue samples (N=50) were determined using a paired *t*-test. (B) Tissue samples from the ONCOMINE database were measured for levels of ADAM9 expression in normal (N=9) and PCa tissue (N=25). (C) Methylation in the ADAM9 promoter region was analyzed using the UALCAN web server. (D) Representative images of IHC staining for ADAM9 in tissue samples from healthy individuals and PCa patients. (E) Tumor tissues were categorized as exhibiting high or low levels of ADAM9 protein expression and significant between-group differences were analyzed by the chi-square test. *P < 0.05, **P < 0.01, ***P < 0.001, ****P < 0.0001.

To further examine the clinical importance of ADAM9, we analyzed ADAM9 levels in PCa tissue samples obtained from the TCGA and ONCOMINE databases. We found high levels of ADAM9 mRNA expression in PCa tissue compared with healthy tissue samples (Fig. 5A&B). Interestingly, the ADAM9 promoter region exhibited low levels of methylation, indicating high transcriptional activity of the *ADAM9* gene in PCa (Fig. 5C). A positive correlation was observed between ADAM9 and WISP-1 mRNA levels in PCa tissues (Pearson correlation: 0.2; P=9.236e-6) (Supplementary Fig. 2A). Histopathological examination of ADAM9 protein levels also revealed higher levels in PCa tissue sections compared with healthy control samples (Fig. 5D&E). This evidence indicates that ADAM9 is overexpressed in PCa and may facilitate PCa aggressiveness.

### ADAM9 blockade inhibits PCa growth and metastasis in the orthotopic model

**Figure 6:**
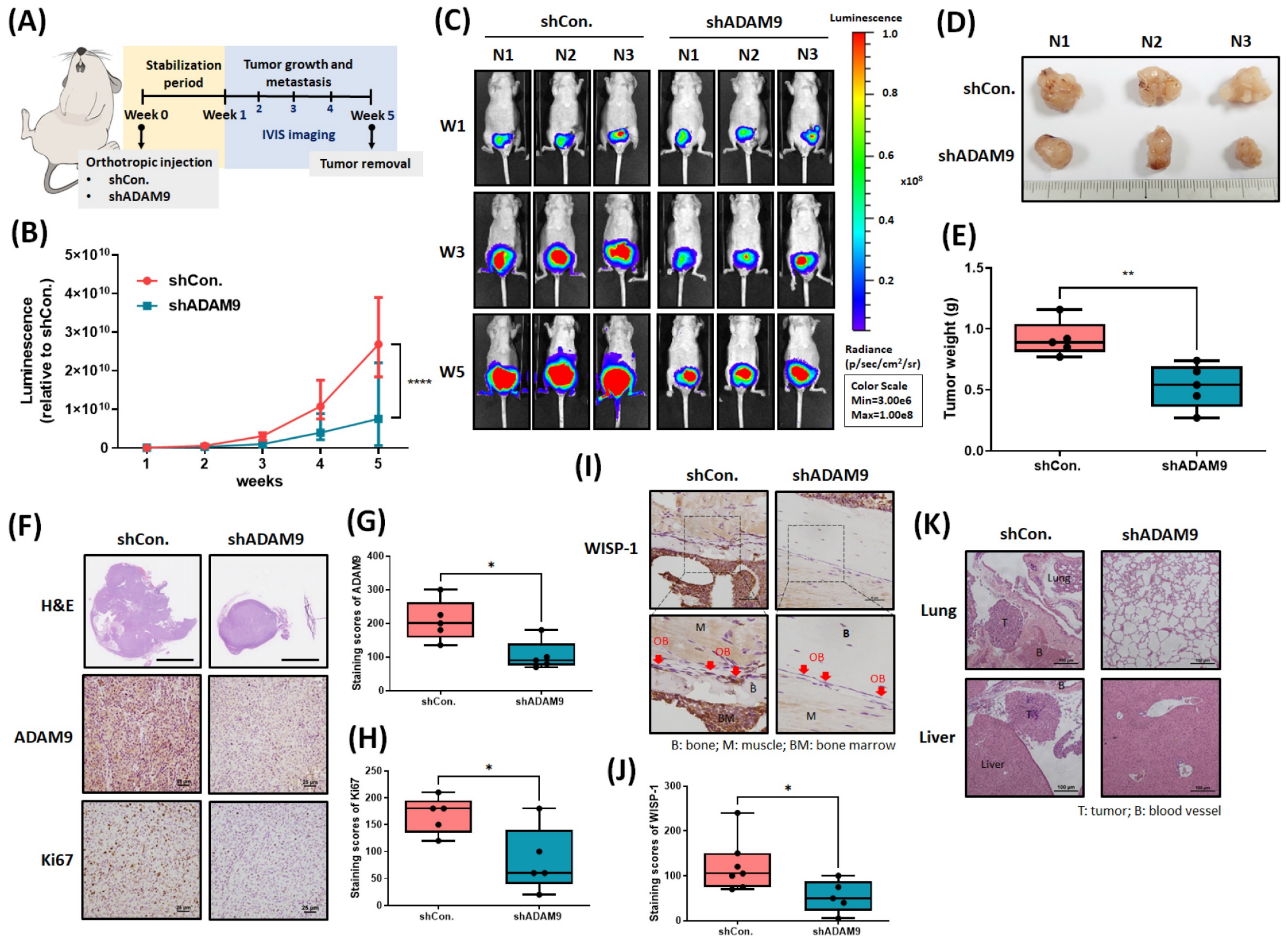
**Targeting ADAM9 prevents PCa growth and metastasis *in vivo*.** (A) The flowchart shows how to establish an *in vivo* model of orthotopic PCa mice. (B&C) Luminescence imaging of tumor-bearing mice was performed weekly using the IVIS spectrum system, and the luminescent intensity of photons emitted from each tumor was quantified. (D) Bright-field imaging of excised tumor tissue. (E) Tumor weights were recorded manually. (F-H) Tumor sections were stained with H&E via whole-mount imaging. IHC staining exhibited ADAM9 and Ki67 expression in tumor tissues. (I&J) IHC staining revealed WISP-1 expression in OBs of tibia specimens. Red arrows: OBs. (K) H&E staining was used to examine liver and lung metastases. *P < 0.05, **P < 0.01, ***P < 0.001, ****P < 0.0001.

Next, we addressed whether ADAM9 blockade enhances the therapeutic efficacy of PCa in tumor xenograft mouse models. First, we implanted PC-3 cells stably expressing pLenti CMV V5-Luc into nude mouse anterior prostates, then sacrificed the mice 5 weeks later (Fig. 6A). ADAM9 blockade significantly suppressed tumor growth, according to IVIS imaging (Fig. 6B&C) and manual tumor weight measurements (Fig. 6D&E). IHC staining demonstrated that ADAM9 blockade significantly suppressed levels of ADAM9 expression and of the proliferation marker protein Ki67 (Fig. 6F-H). Importantly, ADAM9 blockade also enhanced the reduction of WISP-1 expression in PCa-OBs, indicating that ADAM9 depletion in PCa interrupted the cooperation between primary PCa and PCa-OBs (Fig. 6I&J) and prevented distant metastases in the lung and liver (Fig. 6K). These results support the targeting of ADAM9 for reducing the development of metastatic PCa.

## Discussion

The present study determined that primary PCa cells cooperate with distal PCa-OBs to constitute a vicious cycle that leads to metastatic PCa. shRNA-mediated depletion of ADAM9 in primary PCa cells suppressed the formation of PCa-OBs in bone, as well as PCa tumor growth and distal metastasis in an orthotopic PCa mouse model. ADAM9 blockade prevented this vicious cycle, suggesting that this may be a beneficial therapeutic strategy in response to metastatic PCa progression.

Tumor-derived factors and extracellular vesicles predominantly support the creation of the tumor microenvironment (TME) and promote tumor development 28. Numerous nonmalignant cells, including fibroblasts, mesenchymal stem cells, adipocytes and immune cells, are recruited into the TME and are educated to work for the benefit of cancer cells, leading to immunosuppression, malignant transformation and distal metastasis 29-31. Our results indicate that PCa tumors not only influence surrounding nonmalignant cells, but also act in concert with distant OBs located on the bone surface, encouraging metastatic PCa progression. After analyzing 37 candidates from a human cytokine array, we found that PCa-released ADAM9-S is a key regulator that contributes to the transformation of distal OBs into PCa-OBs with WISP-1 overexpression. ADAM9 can present as either a membrane-anchored or soluble form and is responsible for proteolysis of functional proteins, extracellular matrix degradation and cell-cell interactions 32, 33. For example, ADAM9 cleaves the latency-associated peptide to active transforming growth factor β1 (TGF-β1), which subsequently promotes T helper 17 (Th17) cell differentiation 34. TGF-β1 is known to play critical roles in matrix production and osteoblast differentiation 13, 35 and is an important upstream mediator that increases WISP-1 expression 36. Whether TGF-β1 is involved in the ADAM9-induced mediation of WISP-1 levels in PCa-OBs is worth further study. Marked upregulation of ADAM9 in cancer cells and tissues is associated with several different cancers, such as lung, prostate, breast, and brain cancers 37. In triple-negative breast cancer (TNBC), higher ADAM9 expression correlates significantly with poorer outcomes, while inhibition of ADAM9 expression lowers TNBC cell aggressiveness by suppressing AKT/NF-κB signaling 38. In androgen-independent PCa, ADAM9 can form a complex with *N*-α-acetyltransferase 10 protein to maintain ADAM9 protein stability and thus promote tumor cell invasiveness and *in vivo* tumor progression 39. ADAM9 also serves as a significant and independent prognostic marker associated with prostate-specific antigen relapse-free survival in PCa patients treated with ADT 40. In clinical PCa specimens, higher ADAM9 protein expression has been found in malignant prostatic hyperplasia (Gleason scores 6 and 7) compared with benign prostatic tissues 41. ADAM9 expression is even elevated in PCa tissue with low metastatic potential (M1 stage) compared with nonmetastatic (M0) tissue samples 42. Notably, enhanced expression levels of ADAM9-S have been detected in human PCa serum 42, suggesting that ADAM9 may be a predictive biomarker of cancer progression in PCa clinical and serum specimens. This evidence supports the important impact of ADAM9 upon tumor progression.

IMGC936 (an anti-ADAM9 antibody drug conjugate developed by MacroGenics) is undergoing Phase 1 investigations involving patients with TNBC, nonsquamous non-small cell lung cancer, colorectal cancer, gastroesophageal cancer, or pancreatic cancer (NCT04622774). Identifying novel ADAM9 inhibitors from cell-based assays may encourage the design of more selective compounds that help to block ADAM9-induced promotion of cancer 43. According to our study results revealing protumor functions of ADAM9 in PCa, IMGC936 and other novel pharmacological agents that inhibit ADAM9 should be explored in preclinical investigations and clinical trials with PCa patients.

The initiation of metastasis requires tumor cells to exhibit an invasive ability, which is enabled by the development of the EMT process in cancer cells 44, 45. In this study, we provide evidence in support of cooperation between primary PCa and PCa-OBs caused as a result of EMT activation in PCa cells. PCa-OBs expressing WISP-1 increased the levels of N-cadherin and Twist expression, with a concomitant loss of E-cadherin, increasing the aggressiveness and metastatic ability of PCa cells via EMT activity. Similarly, WISP-1 exhibits a powerful impact on activating EMT via the miR-153-3p/Snail axis in oral squamous cell carcinoma 21, or through stimulating AKT and MEK/ERK signaling pathways in melanoma 20. Targeting WISP-1 expression may therefore prevent the development of EMT-related tumor aggressiveness. An intriguing observation from our study is that WISP-1 upregulated both total Twist protein and nuclear translocation expression. However, levels of Twist nuclear translocation are not necessarily dependent upon increases in total Twist protein. Whereas some research has shown that Hsp90β elevates levels of Twist expression by increasing Twist protein stability that ultimately influence Twist translocation from the cytoplasm to the nucleus in hepatocellular carcinoma 46, other research has reported that antiapoptotic Bcl-2 switches on Twist nuclear localization signals and results in the transcriptional activation of EMT under hypoxic conditions 47. Studies have shown that EMT can be induced by ADT in metastatic PCa, so EMT is capable of conferring an invasive potential in tumour epithelial cells 48, 49. In other research, CD44^+^ cancer stem-like cells can switch to EMT cells via TGFβ1-CD44 signaling and drive PCa metastasis 50. Our findings are consistent with these reports, showing that EMT functions have a marked, comprehensive influence upon metastatic PCa progression. Notably, an ongoing neoadjuvant Phase 2 study including patients with PCa is comparing the effects of ADT with or without chemotherapeutic agents against SRC or MEK (degarelix, enzalutamide, trametinib, or dasatinib), by evaluating the inhibition of mesenchymal markers N-cadherin and vimentin (NCT01990196). Numerous clinical trials are investigating EMT therapeutic approaches in combination with known cancer-targeted therapies, immunotherapies, and/or novel compounds 51.

Since PCa-derived factors are favorable for OB differentiation and mineralization in the bone microenvironment, PCa bone metastases are mainly characterized as osteoblastic (forming new bone), according to radiographic and/or pathologic appearances of the lesions 52. Similarly, we found that primary PCa cells influence the transformation of OBs into PCa-OBs, with subsequent increases in the release of WISP-1 from PCa-OBs in the bone microenvironment. However, we did not evaluate whether PCa-OBs expressing WISP-1 regulate cancer-related osteoblastogenesis. This aspect deserves further investigation. Transgenic mice overexpressing human WISP-1 in preosteoblasts reportedly exhibit enhanced bone mineral density compared with their wild-type controls, which suggests that WISP-1 positively regulates osteoblastogenesis *in vivo* 53. In addition, it appears that PCa bone metastases require both osteoclastic and osteoblastic activity 54. Confirmation is needed as to whether primary PCa influences distal OBs and osteoclasts in bone to advance its own malignant characteristics.

## Conclusion

**Figure 7:**
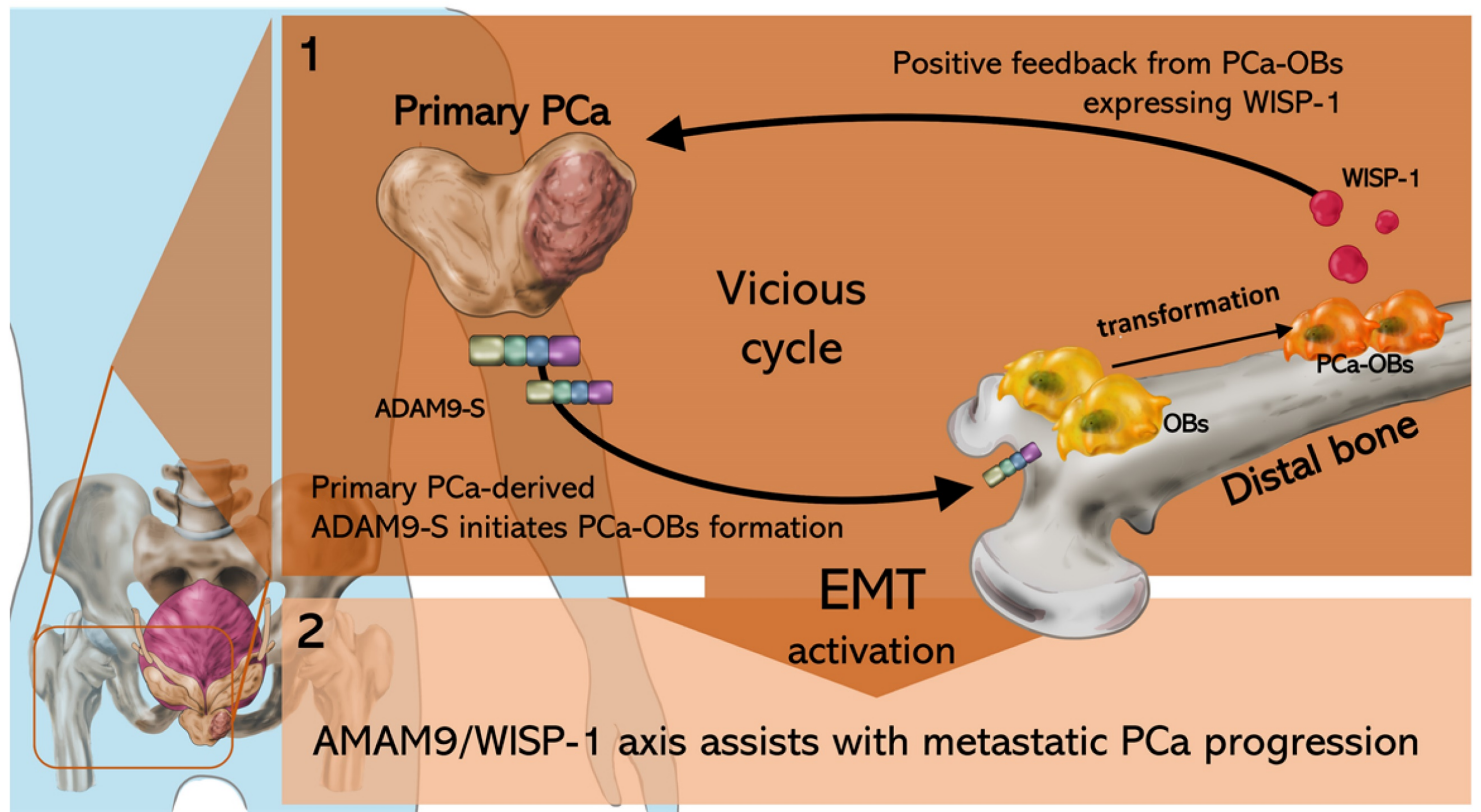
The schematic diagram shows how the ADAM9/WISP-1 system creates a vicious cycle between primary PCa and OBs that stimulates metastatic PCa progression.

In conclusion, our study demonstrated that the ADAM9/WISP-1 axis triggers primary prostate tumor growth and distant metastasis by cooperating with OBs in bone. Interfering with ADAM9 expression in primary PCa interrupts the formation of the vicious cycle between primary PCa cells and OBs, impairing PCa-OB formation, tumor growth and metastatic spread (Fig. 7).

## Supplementary Material

Supplementary materials and methods, figures.

## Abbreviations

ADAM9: ADAM metallopeptidase domain 9
ADT: androgen deprivation therapy
ALP: alkaline phosphatase
BMP: bone morphogenic protein
CM: conditioned medium
ELISA: enzyme-linked immunosorbent assay
EMT: epithelial-mesenchymal transition
H&E: hematoxylin and eosin
HIF-1α: hypoxia-inducible factor 1-alpha
IHC: immunohistochemistry
LHRH: luteinizing hormone-releasing hormone
OBs: osteoblasts
OPN: osteopontin
PCa: prostate cancer
PSA: prostate-specific antigen
qRT-PCR: quantitative real-time polymerase chain reaction
TCGA: The Cancer Genome Atlas
TME: tumor microenvironment
TNBC: triple-negative breast cancer
VEGF-A: vascular endothelial growth factor A
WISP-1: WNT1 inducible signaling pathway protein 1
