# Supplementary material for: The ADAM9/WISP-1 axis cooperates with osteoblasts to stimulate primary prostate tumor growth and metastasis
Source: Int J Biol Sci. 2023 Jan 1;19(3):760–71. doi: 10.7150/ijbs.77495 (PMC9909997; doi:10.7150/ijbs.77495)
Supplement: Supplementary file 1 — Supplementary materials and methods, figures. [file ijbsv19p0760s1.pdf]

The ADAM9/WISP-1 axis cooperates with osteoblasts to stimulate primary prostate tumor growth and metastasis

An-Chen Chang, Liang-Wei Lin, Yen-Chen Chen, Po-Chun Chen, Shan-Chi Liu, Huai-Ching Tai, Hsi-Chin Wu, Shian-Ying Sung, Tien-Huang Lin, Chih-Hsin Tang

This file includes:

Materials and Methods

Supplementary Data

Materials and Methods

Cell lines

The human osteoblastic-like cell line MG-63 was obtained from the Bioresource Collection and Research Center (BCRC, Hsinchu, Taiwan). The human prostate epithelial cell line PZ-HPV-7 and human prostate cancer cell lines (LNCaP, PC3 and DU145) were obtained from the American Type Culture Collection (ATCC, VA, USA).

Western blot analysis

Electrophoresis and transfer methods were the same as those described in our previous study [1]. In brief, membranes were incubated with primary anti-ADAM9 (R&D system, MN, USA), WISP-1 (Abcam, Cambridge, UK), E-cadherin (Cell Signaling, MA, USA), vimentin (Cell Signaling, MA, USA), N-cadherin (Cell Signaling, MA, USA), Twist (Cell Signaling, MA, USA), and anti-β-actin (Merck, Darmstadt, Germany) antibodies at 4°C overnight. After three washes with PBS, membranes were incubated with the appropriate horseradish peroxidase-conjugated secondary antibody (Cell Signaling, MA, USA) at 37°C for 1 h. Immunoblots were imaged using a ChemiDoc-It Imaging System (UVP Inc., CA, USA).

Immunohistochemistry (IHC)

Tumor tissues were stained with primary antibodies (Ki67, ADAM9, or WISP-1), according to the method described in our previous study [2]. IHC tissues were grouped into categories of low and high staining, scored by taking into account the percentage of positive detection (0 to 100) and the intensity of the staining (0, negative; 1, weak; 2, moderate; 3, strong), providing a final score ranging from 0 to 300. Levels of ADAM9 protein expression were categorized by IHC staining as either low (0–150) or high (151–300).

Patient serum and tissue preparation

Human PCa tissue arrays were purchased from US Biomax (MD, USA). Serum samples were collected from 11 patients undergoing surgical resection in China Medical University Hospital, under approval granted by the Institutional Review Board of China Medical University Hospital (CMUH109-REC2-142). Written informed consent was obtained from each study participant before enrollment. WISP-1 protein levels in patient serum were detected using the WISP-1 ELISA kit (PeproTech, NJ, USA).

1. Chang, A.C., et al., *WISP-1 Promotes Epithelial-Mesenchymal Transition in Oral Squamous Cell Carcinoma Cells Via the miR-153-3p/Snail Axis.* Cancers (Basel), 2019. **11**(12).
2. Chang, A.C., et al., *Osteoblast-secreted WISP-1 promotes adherence of prostate cancer cells to bone via the VCAM-1/integrin alpha4beta1 system.* Cancer Lett, 2018. **426**: p. 47-56.

Supplementary Data

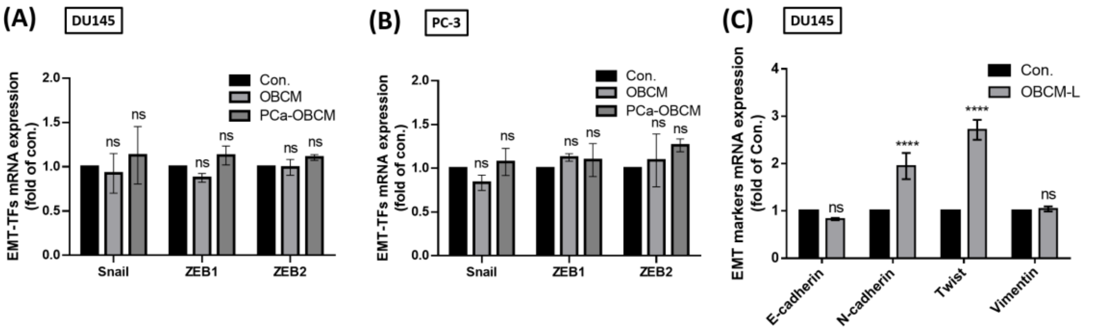

Supplementary Figure 1. The mRNA expression levels of EMT markers and EMT-TFs in PCa cells. (A&B) OBCM and PCa-OBCM were added to PCa cells for 24 h, then examined for Snail, ZEB1, and ZEB2 mRNA expression by qRT-PCR assays. (C) Treatment of PCa cells with OBCM-L for 24 h. E-cadherin, N-cadherin, Twist, and vimentin mRNA expression were analyzed by qRT-PCR assays. *P < 0.05, **P < 0.01, ***P < 0.001, ****P < 0.0001.

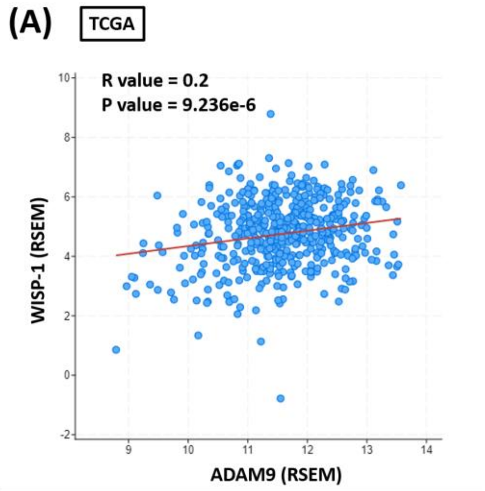

Supplementary Figure 2. Positive correlation between ADAM9 and WISP-1 in human PCa. (A) The mRNA levels of ADAM9 and WISP-1 in PC tissues were analyzed via cBioPortal according to TCGA-PRAD dataset. R value is analyzed from Pearson correlation coefficient.
